# The effect of the Family Health Strategy on usual source of care in Brazil: data from the 2013 National Health Survey (PNS 2013)

**DOI:** 10.1186/s12939-016-0440-7

**Published:** 2016-11-17

**Authors:** Inês Dourado, Maria Guadalupe Medina, Rosana Aquino

**Affiliations:** Institute of Collective Health/Federal University of Bahia, Brazil. Rua Basílio da Gama, s/n, Campus Universitário do Canela, 40110-040 Salvador, Bahia Brazil

**Keywords:** Usual source of care (USC), Primary health care, Family health strategy, National health survey, Brazil

## Abstract

**Background:**

A usual source of care (USC) has been conceptualized as having a health provider or place available for patients to consult when sick or in need of medical care. Having a USC is a means to achieve longitudinality of care with Primary Health Care (PHC) providers. Brazil has made enormous progress in PHC and thus provides an important opportunity to investigate USC in a middle-income country context.

**Methods:**

This study uses data from a nationally representative household survey, the 2013 National Health Survey (*n* = 62,986), to describe the prevalence of having a USC in Brazil and to investigate to what extent the Family Health Strategy (FHS) has contributed to USC prevalence. Analyses include descriptive, bivariate and multivariable Poisson regression.

**Results:**

Show very high rates of people reporting any type of USC (74.4 %) and more than one third reporting PHC as their USC. Household enrolment in the FHS was positively associated with having any USC (PR:1.09; 95 % CI: 1.07–1.12) and a stronger association with having PHC as the regular source of care (PR:1.63;95 % CI:1.54–1.73). FHS enrolment was negatively associated with reporting emergency/urgent care facilities as one’s USC (PR: 0.67; 95 % CI: 0.59–0.76). The association between the more consolidated FHS with having a USC was strongest in the poorest regions of the country (North, Northeast and Central-West). Having PHC as one’s USC showed a positive dose-response relationship with the FHS in all regions, especially in the Central-West.

**Conclusions:**

Our results have important implications for the health care model in Brazil and in other countries, especially those seeking to base their national health systems more strongly on primary health care. The study suggests expanding primary health care can increase the establishment of a USC which can help assure better monitoring of chronic conditions and attention to patient needs.

## Background

A usual source of care (USC) has been conceptualized as having a specific health provider or place for patients to consult when sick or in need of medical care [1]. It is directly related with longitudinality, one of the core dimensions of primary health care [2], defined by patient follow-up over time by a general practitioner or PHC staff, characterizing an implicit therapeutic relationship based on professional responsibility and mutual confidence [3]. Furthermore, it has been shown to have beneficial effects on health care utilization and outcomes, and reduces unnecessary referrals to specialists [4–6].

Barbara Starfield argues that having a USC requires longitudinality of care with a PHC provider [6–9], despite other services that may substitute for a USC, such as specialist physicians or the emergency room. Lack of adequate access to PHC and/or acute exacerbation of a chronic condition may be explanations for reporting emergency services as a USC for some populations. Many studies have reported the increase in emergency services, even in high-level income countries [10–12]. A recent exploratory study in England shows that more than 25 % of non-planned accident and emergence services consultations are related to difficulties in obtaining a general practice appointment [13]. Furthermore, the use of emergency services as a USC is less likely to resolve the patient’s overall health needs and may lead to overcrowded emergency services, increased risk of nocosomial infections, and unnecessary expenses for the individual and the health system [14, 15].

Brazil provides an important location to investigate USC as a middle-income country that has made enormous progress in PHC and in overcoming inequities in the last decade, but remains with major inequalities between regions, communities and population groups [16–19]. Since the 1990s, Brazil’s health system has been aiming to achieve universality and comprehensiveness of care. One of the most important changes was the implementation of the Family Health Strategy (FHS) in 1994 with a large expansion from 2000, reaching almost 60 % coverage of the Brazilian population by 2013 [20]. It is now considered the world’s largest community-based PHC program. The beneficial impact of FHS is evidenced by positive evaluations by users, managers and health care professionals [6, 15, 21], improved availability, access to and use of health services [22] and improved health indicators, such as reduced infant mortality, avoidable hospitalization and heart and cerebrovascular disease mortality [23–27]. Despite these positive results, national inequalities and different models and/or insufficient FHS implementation remain [19, 28, 29].

The 2013 Brazilian National Health Household Survey collected indicators of utilization of health service from the user’s point of view, as well as individual data on FHS enrollment providing an opportunity to: describe the prevalence of reporting any type of USC in Brazil; examine prevalence and correlates of the different types of healthcare services reported as USCs; and investigate to what extent the FHS has contributed to the presence and type of USC reported and consequently to what extent FHS reduced inequities in access to health care.

## Methods

The Brazilian National Health Survey (*Pesquisa Nacional de Saúde-PNS*) is nationally-representative household survey developed by the Brazilian Institute of Geography and Statistics (IBGE) and Ministry of Health and conducted in 2013. Main objectives of PNS are: to describe the health situation and lifestyles of the Brazilian population, access and use of health services, and evaluation of the health care and prevention measures provided by the National Public Health System (*Sistema Único de Saúde*-SUS) [30]. The survey employs a complex sampling design. The primary sampling units are census tracts based on the 2010 census and randomly selected from the IBGE national master sampling plan. Within each census tract households were randomly selected from a national registry of addresses. Within selected households a randomly selected respondent aged 18 or over was invited to take part in the study. In order to account for losses the estimated sample size was of around 80,000 households based on a predicted non-response rate of 20 %. At the end of fieldwork, a total number of 81,167 households were visited, of which 69,994 were occupied, with 64,348 household interviews and 62,986 individual interviews with a selected household resident being conducted representing an overall response rate of 78 % [31]. Face to face interviews were conducted with properly trained interviewers and the assistance of handheld computers. No incentives were provided. Person-level survey weights take into account the probability of selection as well as non-response rates. Further detail of sample size calculations and weighting procedures can be found in Souza Jr et al, 2013 [32]. There are no missing data in the PNS as IBGE impute data for missing information. The PNS project was approved by the National Commission of Ethics in Research (CONEP) in June 2013, Regulation No. 328.159. The PNS data are publicly available on the IBGE and Fiocruz websites. The data do not have identification of the participant and the lowest level of available geographical breakdown is capital, metropolitan belt, rest of the state, which are too large to identify a participant.

The PNS data include general information on all residents of the household (given by one of the household residents who could inform about the socioeconomic situation and health of all of its residents) and from a randomly selected resident aged 18 or over. Further details about the PNS development have been reported in [31] and [32].

USC and its types were defined from two questions: “Do you usually go to the same place, the same doctor or health service when you need health care (yes or no) and “When you are sick or need health care where do you usually go”. Then, we constructed 6 outcomes: 1- Any USC- coded “no” for those who said no to the first question or go to pharmacies (no USC) and “yes” for those who go to public or private health centers, public or private home care, public or private hospitals and emergency care. 2- No USC (the opposite of having a USC); 3- Only PHC Provider-coded “no” if no USC or all sources except PHC and “yes” for PHC or home care provided by PHC; 4-Only Private Provider- coded “no” if no USC and all sources except private providers or “yes” for those who goes to outpatient private health centers or private home care; 5-Not an emergency care-coded “no” if no USC and other emergency room/urgent care or “yes” for all sources except emergency room/urgent care; 6-Only Emergency room/urgent care facility- coded “no” if no USC or all sources except emergency room/urgent and “yes” for all sources of emergency room/urgent care). The main “exposure” is whether the respondent’s household is registered as enrolled in the FHS. Of the total Brazilian population covered by the FHS (54 %), 86.6 % were registered over 1 year and 70 % had regular visits by community health workers (CHW) in the last year. In the construction of the FHS enrolment we consider 1- not enrolled; 2- incipient (those who were enrolled for less than 1 year or did not have regular visits by CHW in the last year) and 3-consolidated (those enrolled for 1 year or more and with regular visits by CHW in the last year). The adopted definition sought to address not only the time of implementation of the FHS, but a “proxy” (regular CHW visits) for systematic monitoring by the FHS team to the survey participant’s family. Other covariates (potential confounders) were used to adjust expected differences in prevalence rates of reporting a USC compared to not reporting a USC. And included: type of health care coverage- a combination of FHS and private insurance enrollment with four categories (1-none; 2- FHS enrolment only; 3- FHS enrolment and private insurance and 4- private insurance only; age; sex; self-reported skin color according to the official Brazilian census categories (white, black, *pardo-someone from a mixture of skin color, that is, a person generated from some miscegenation-* Asian, native Brazilian/Indigenous); educational attainment (none, less than high school completed, high school completed, more than high school); geographical area of residence (rural versus urban); state capital residence (versus elsewhere); country region (North, Northeast, South, Southeast, Central- West); self reported health status (excellent/very good/good versus fair/poor); self report of a chronic disease; and comorbidity (reporting of two or more chronic). We present descriptive statistics and bivariate analyses of USC and its different types by study covariates with Pearson chi square tests and respective *p* values to test for independency. We then present results of multivariable Poisson regression models for each USC outcome as the prevalence is over 10 % and adjusted prevalence ratios and 95 % confidence intervals (CI) for the association of enrollment in the FHS and USC and were estimated. Country region was used as “proxy” to the measurement of inequality in evaluating the association of FHS enrollment with USC. Historically, Brazil has marked geographical/regional inequalities such as social economical development, income distribution and distribution of public resources such as transportation, sanitation, health and educational services. Several studies demonstrate secular differences that divide the country into poorer (North and Northeast) and richer regions (South and Southeast) [19, 33, 34]. All analyses were performed using Stata version 12.1 and results incorporate appropriate weights and control for the complex sample design.

## Results

Descriptive and bivariate statistics for the sample are shown in Table 1. Slightly more than half of our sample was female, approximately one-fifth was 25–34 years of age and 12.3 % was 65 and older. About half of respondents self-classified as white and 41.9 % as *pardo*. Up to 50 % completed primary school. Most (86.2 %) resided in urban areas, a quarter lived in one of the 26 state capitals or federal district and 43.8 % were from the Southeast Region. The majority (74.4 %) of the sample reported having any type of USC but differed according to demographics. Compared to those without a USC, having a USC predominated among women, the elderly (≥65 years), those who self-identify as white, among extremes of educational attainment (less than primary school or completed college), more likely to reside in urban areas, other cities than the capital and in the South and Southeast Regions. Regarding types of USC separately: 61.9 % reported a source other than emergency/urgent care services, 35.5 % only PHC, 17.5 % only private physician and 12.6 % only emergency care. Having PHC as an USC was slightly higher among women; among those 44 and older; those self-classified as *pardo* or indigenous; those with none or educational less than primary school; rural residence; cities other than the capital and in the South Region. Other types of USC and demographics are in Table 1.

**Table 1:** Descriptive and bivariate analysis of the population demographics by percentages of usual source of care (USC)

	Usual source of care *N* = 60.202				Types of usual source of care *N* = 60.202							
Characteristics	Total	Any (% of total)	None (% of total)	χ^2^ *p* value	Primary Health Care provider (% of total)	χ^2^ *p* value	Private Provider (% of total)	χ^2^ *p* value	Not emergency care (% of total)	χ^2^ *p* value	Emergency room/urgent care	χ^2^ *p* value
Total		74.45	25.55		35.54		17.47		61.88		12.57	
Demographic												
Sex												
Female	52.9	76.09	23.91	0.00	36.74	0.00	17.92	0.10	63.38	0.00	12.70	0.61
Male	47.1	72.61	27.39		34.21		16.97		60.18		12.43	
Age												
18–24	15.93	72.31	27.69	0.00	36.34	0.01	13.05	0.00	58.47	0.00	13.85	0.00
25–34	21.63	72.47	27.53		33.21		16.22		58.55		13.92	
35–44	19.19	74.74	25.26		35.27		18.35		62.09		13.65	
45–54	17.5	75.09	24.91		36.44		18.34		63.17		11.92	
55–64	13.46	76.29	23.71		36.99		19.59		65.29		11.00	
> = 65	12.29	77.32	22.68		36.20		20.51		66.24		11.09	
Race/skin color												
White	47.46	75.95	24.05	0.00	31.20	0.00	23.95	0.00	62.10	0.64	13.84	0.00
Black	9.2	72.82	27.18		38.31		11.30		60.94		11.88	
Asian	0.94	72.26	27.74		22.66		27.86		57.14		15.11	
*Pardo*	41.98	73.19	26.81		40.08		11.32		61.93		11.26	
Indigenous	0.42	72.48	27.52		41.86		12.33		62.24		10.24	
Education attainment												
None/illiterate	13.69	73.42	26.58	0.00	47.25	0.00	6.34	0.00	64.20	0.00	9.21	0.00
Less than primary	25.25	76.59	23.41		47.19		8.91		66.12		10.47	
Primary complete	9.92	73.94	26.06		38.15		12.16		59.85		14.09	
HS- incomplete	5.61	73.39	26.61		40.19		12.20		61.53		11.85	
HS - complete	28.04	73.21	26.79		31.31		19.23		58.66		14.55	
College incomplete	4.77	72.29	27.71		19.94		30.03		56.49		15.80	
College complete	12.74	75.73	24.27		10.96		44.32		61.79		13.94	
Geographical area of residence												
Urban	86.21	74.70	25.30	0.11	33.36	0.00	19.44	0.00	61.03	0.00	13.67	0.00
Rural	13.79	72.89	27.11		49.22		5.22		67.19		5.71	
State capital residence												
No	75.27	75.49	24.51	0.00	38.99	0.00	14.84	0.00	63.61	0.00	11.88	0.00
Yes	24.73	71.28	28.72		25.07		25.50		56.60		14.68	
Country Region												
North	7.44	68.25	31.75	0.00	37.48	0.00	10.01	0.00	57.37	0.00	10.87	0.00
Northeast	26.62	69.58	30.42		37.20		10.15		59.98		9.59	
Southeast	43.79	77.71	22.29		32.62		21.78		61.88		15.83	
South	14.78	78.49	21.51		41.29		21.88		68.32		10.17	
Central-West	7.36	70.84	29.16		33.45		17.05		60.33		10.51	

*P* values from Pearson χ^2^ tests

Table 2 presents descriptive and bivariate statistics for the respondents’ reported health variables. Most reported good self-rated health, slightly more than one third reported a chronic disease, few reported comorbidities (12.7 %), approximately one-fourth reported enrollment in private insurance, more than half reported enrollment in the FHS (20.4 % incipient and 34.2 % consolidated FHS coverage). Regarding type of health care, the majority of respondents were enrolled in the FHS by itself. Compared to those without a USC, having a USC was more common among those with chronic conditions, with comorbidities, enrolled in private insurance, enrolled in the FHS (even higher with consolidated FHS coverage), and among those with FHS by itself or in combination with private insurance. Having PHC as one’s USC was higher among those reporting poor health, chronic disease, comorbidity, not enrolled in private insurance, and enrolled in the FHS.

**Table 2:** Descriptive and bivariate analysis of the health variables by percentages of usual source of care (USC)

	Usual source of care *N* = 60.202				Types of usual source of care *N* = 60.202							
Characteristics	Total	Any (% of total)	None (% of total)	χ^2^ *p* value	Primary Health Care provider (% of total)	χ^2^ *p* value	Private Provider (% of total)	χ^2^ *p* value	Not emergency care (% of total)	χ^2^ *p* value	Emergency room/urgent care	χ^2^ *p* value
Health Variables												
Self-Report of Health Status												
Fair/poor	32,24	74,68	25.32	0.60	42,14	0.00	11,52	0.00	64,18	0.00	10,50	0.00
Excellent/very good/good	67,76	74,34	25.66		32,41		20,31		60,78		13,56	
Reporting of chronic disease												
No	64,66	72,21	27.79	0.00	34,60	0.00	16,32	0.00	59,60	0.00	12,62	0.82
Yes	35,34	78,55	21.45		37,27		19,58		66,05		12,50	
Reporting of two or more chronic												
No	87,29	73,60	26.40	0.00	35,12	0.00	17,24	0.02	61,19	0.00	12,41	0.08
Yes	12,71	80,29	19.71		38,50		19,09		66,60		13,68	
Enrolled in private insurance (PI)												
No	73,6	73,18	26.82	0.00	44,95	0.00	6,54	0.00	61,86	0.96	11,31	0.00
Yes	26,4	78,01	21.99		9,33		47,96		61,91		16,09	
Enrolled in the Family Health Strategy (FHS)												
No	45,39	71,62	28.38	0.00	23,18	0.00	24,86	0.00	56,11	0.00	15,51	0.00
Yes	54,61	76,80	23.20		45,82		11,34		66,67		10,13	
Level of enrollment FHS												
Not enrolled	45,39	71,62	28.38	0.00	23,18	0.00	24,86	0.00	56,11	0.00	15,51	0.00
Incipient	20,41	75,23	24.77		40,20		13,96		63,33		11,89	
Consolidated	34,20	77,74	22.26		49,18		9,77		68,65		9,08	
Type of health care coverage												
No FHS, no PI	29,09	68,09	31.91	0.00	33,55	0.00	9,44	0.00	53,46	0.00	14,62	0.00
Only FHS	44,51	76,50	23.50		52,40		4,65		67,35		9,15	
FHS and PI	10,10	78,11	21.89		16,84		40,80		63,63		14,48	
Only PI	16,30	77,94	22.26		4,67		53,39		60,85		17,09	

*P* values from Pearson χ^2^ tests; FHS = Family Health Strategy; PI = private health insurance; Incipient FHS = enrollment for less than 1 year or did not have regular visits by community health worker in the last year; Consolidated FHS = enrollment for 1 year or more and with regular visits by community health worker in the last year

Tables 1 and 2 shows similar comparisons but in the opposite direction for those who did not report a USC as this variable is the complement of having a USC.

Table 3 presents results of the multivariable Poisson regression models for any USC and each USC type. Levels of FHS consolidation showed an overall positive association with having any USC, but of a small magnitude. FHS was more strongly associated with having PHC as one’s USC with a dose response relationship: consolidated FHS had a higher likelihood of having PHC as one’s USC, less so for incipient FHS coverage. Levels of FHS consolidation showed a negative association with having a private physician as one’s USC. Levels of FHS consolidation showed a positive dose-response association with having any service except emergency/urgent care service as one’s USC. And levels of FHS consolidation showed a negative association with reporting emergency/urgent care services as one’s USC with a strong dose response relationship.

**Table 3:** Prevalence ratios (PR) from poisson regression models for the association between FHS enrollment and USC, stratified by Country Region. Brazil 2013

Usual source of care															
	Any USC			Primary Health Provider			Private Provider			Not emergency care			Emergency room/urgent care		
Level of FHS*** enrollment	PR^a^^*^	95 % CI		PR*	95 % CI		PR*	95 % CI		PR*	95 % CI		PR*	95 % CI	
Incipient (vs not enrolled)	1,06	1,03	1,09	1,45	1,36	1,55	0,85	0,79	0,92	1,13	1,09	1,17	0,81	0,71	0,91
Consolidated	1,09	1,07	1,12	1,63	1,54	1,73	0,74	0,68	0,81	1,20	1,17	1,24	0,67	0,59	0,76
By Country Region**															
North															
Incipient (vs not enrolled)	1,07	1,00	1,15	1,35	1,18	1,55	0,73	0,58	0,90	1,14	1,05	1,25	0,75	0,59	0,96
Consolidated	1,11	1,02	1,20	1,45	1,27	1,67	0,54	0,42	0,70	1,18	1,07	1,31	0,76	0,55	1,04
Northeast															
Incipient (vs not enrolled)	1,12	1,06	1,18	1,45	1,30	1,61	0,91	0,78	1,06	1,14	1,06	1,22	1,05	0,83	1,33
Consolidated	1,19	1,14	1,24	1,76	1,60	1,93	0,74	0,61	0,90	1,28	1,20	1,34	0,81	0,66	1,00
Southeast															
Incipient (vs not enrolled)	1,03	1,00	1,07	1,46	1,30	1,63	0,80	0,70	0,92	1,10	1,05	1,18	0,77	0,64	0,92
Consolidated	1,07	1,03	1,12	1,61	1,45	1,79	0,80	0,69	0,93	1,18	1,12	1,25	0,70	0,58	0,86
South															
Incipient (vs not enrolled)	1,07	1,03	1,13	1,49	1,32	1,68	0,90	0,80	1,03	1,13	1,05	1,21	0,80	0,59	1,09
Consolidated	1,08	1,02	1,15	1,63	1,44	1,85	0,66	0,54	0,81	1,15	1,06	1,25	0,71	0,48	1,03
Central-West															
Incipient (vs not enrolled)	1,11	1,04	1,18	1,67	1,45	1,91	0,86	0,71	1,05	1,15	1,06	1,25	0,93	0,72	1,20
Consolidated	1,16	1,10	1,22	1,93	1,72	2,17	0,80	0,69	0,93	1,25	1,17	1,34	0,70	0,55	0,89

^a^Comparing those that reported a USC with those that did not report

*Adjusted for age, sex, race/skin color, education attainment, geographical area of residence, state capital residence, country region, self report of health status, reporting of chronic disease, comorbidity, enrolled in private insurance

**Adjusted for age, sex, race/skin color, education attainment, geographical area of residence, state capital residence, self report of health status, reporting of chronic disease, comorbidity, enrolled in private insurance

***Family Health Strategy

Table 3 also shows results of the multivariable Poisson regression models for any USC and each USC type by region. The association between levels of FHS consolidation and having a USC was positive and stronger in the poorest regions of the country (the North, Northeast and Central West). Having PHC as one’s USC showed a positive dose response relationship in all Regions especially in the Central-West. Having a private physician as one’s USC was negatively associated with levels of FHS consolidation and was stronger in the South Region. Reporting any service except emergency/urgent care as one’s USC was positively associated with levels of FHS consolidation and stronger in the Central West region. In addition, having emergency/urgent care services as one’s USC was negatively associated (in a dose response manner) in the Southeast but did not reach levels of statistical significance in the other regions.

## Discussion

This study reports very high rates of having a USC - only one quarter of the Brazilian population did not report a USC. Most Brazilians do not rely on emergency/urgent care services as their USC. More than one third report PHC as their USC and a small proportion report their USC as private physicians or emergency/urgent care facilities. It is well documented that health services utilization depends on individual needs determined by demographic and social characteristics in addition to health status [35], and on the accessibility, acceptability, and appropriateness of health services as determined by the health system’s means of healthcare organization, financing, and delivery.

Despite FHS consolidation as the main PHC organization model in Brazil, its implementation is heterogeneous and could explain why only one third reported PHC as their usual source of care. Qualitative and quantitative studies have demonstrated differences in FHS performance between states, municipalities and even within a single municipality geographical area [36, 37]. This heterogeneity reflects geographical inequalities and differences in the implementation across the country. Another possible explanation is that the implementation of the universal public health system in Brazil is relatively recent compared to other developed countries. And weaknesses in primary health care remains as an important challenge for managers and researchers [21].

In Brazil, the national public health system (the *SUS*)--especially the consolidation of primary health care through the FHS--has been associated with beneficial effects on equity of access to healthcare [19] and has been shown to provide a strong link between users and FHS health care teams. This link has been carefully constructed through a process of territorialization and other mechanisms such as the presence of CHW in the teams to strengthen links between the population and the health system. Territorialization means each multi-professional health team (composed of a physician, a nurse, a nurse assistant and 4–6 CHWs) is assigned a specific territory and has a list of which families it serves. Teams are organized by local geographic areas to provide primary care to about 1000 families (or approximately 3500 people). Furthermore, one of the CHW tasks is to visit the households in the catchment area regularly especially in homes where there is someone with a chronic condition, a woman who recently gave birth, or a young child.

Having PHC as one’s USC was more pronounced in sub-groups of the population such as women and those 44 and older, as expected, but also among those who often face barriers (inequities) to health care including *pardos* and indigenous people, those with lower levels of educational attainment, those residing in rural areas and those residing in non-capital cities. Most likely, this is the result of FHS consolidation in certain areas. As one would also expect, having any type of USC was more frequent among those with higher health needs, those enrolled in private insurance or those enrolled in the FHS. However, having PHC as one’s USC was more frequent among those not enrolled in private insurance, and among those with higher health needs and those enrolled in the FHS.

The consolidation of the FHS was associated with having PHC as a USC and the more consolidated the FHS the higher the rate of reporting PHC as one’s USC in comparison to those not enrolled in the FHS and after controlling for a set of potential confounders. This result is consistent with a previous study showing that families enrolled in the FHS were more likely to have a usual source of medical care [35]. This previous study conduct in 2008 based on National Household Survey found that adults living in households enrolled in FHS were more likely engaged with a usual source of care as compared to those in families with neither FHS enrollment nor private health plans.

The observed trend was present in all five Brazilian regions. However, it was more pronounced in the Northeast, one of the poorest Regions of the country and in the Central West. In Brazil, inequalities among the population are still very much present and Viacava (2010) analyzing data from 10 years on access and use of health services indicates that access increased significantly in Brazil mainly for those living in the poorest regions of the country [38].

Emergency/urgent care services in most cases do not provide either informational or clinical continuity of care. Patients may seek this type of service due to acute episodes of chronic conditions and/or because of fewer perceived barriers to accessing higher level medical technologies [39]. This study has shown that FHS consolidation was negatively associated with reporting emergency/urgent care services as one’s USC. Further, the more consolidated the FHS coverage, the lower the rate of reporting emergency/urgent care services as one’s USC, even after controlling for a set of potential confounders. This trend was most pronounced in the Southeast and South (the richest regions of the country) and in the Central-West.

It is well known that a positive patient/provider relationship is essential for successful treatment. This applies to improving patient adherence to treatment plans for chronic conditions as well as dealing with stigmatized health problems such as mental health or tuberculosis, all of which require trust as a fundamental ingredient in the therapeutic process. However, trust between patients and health providers needs time to develop. A study conducted in the United Kingdom demonstrates that the length of time of a patient/physician relationship was significantly and independently associated with trust [9]. In our study, consolidated FHS (household enrolled 1 year or more with 2 or more visits from a community health worker in the past year) was associated with having PHC as one’s USC and reporting less use of emergency services as a USC. This finding emphasizes not only the importance of PHC vis-a-vis FHS in Brazil but the consolidation of the Program throughout the country.

Limitations of the study include: 1- the definition of the outcome variable – USC – refers to a provider or place a patient consult when sick or in need of medical advice and is considered one of the hallmarks of primary health care [5]. Furthermore, USC is used in many self-reported surveys and is operationalized through questions such as “Is there one particular place that you go if you are sick or need advice about your health?” and “Is there a regular doctor you usually see at this place?” [8, 9]. In our study USC was defined as in health services studies. While it is true that “see the same doctor” and “go to same place” have different implications regarding longitudinality and continuity of care, Mainous et al., for example, find that trust in one’s physician has more beneficial consequences in effectiveness of medical care than seeing the same provider [7]. In our study, it was impossible to differentiate between having same doctor or the same place since the questionnaire did not make this distinction. However, in Brazil, different from other countries most primary care is provided by only one physician (in the Family Health Strategy, teams are composed by one physician, one nurse and 6 community health agents). There are also PHC teams composed by more than one physician, especially in big cities, related to other types of PHC organization besides FHS. But the consolidation of PHC in Brazil is mainly due for the implementation of FHS. Therefore, the limitation of the questionnaire most likely does not strongly affect longitudinality as a criterion for USC. 2- Results are based on self-report and may represent overestimates of true values of individual reports of a usual source of care. Nevertheless, evidence has shown high levels of USC in other studies and we provide estimates of different types of USC here to provide more valid information in the country as a whole and in different regions. 3- Because the data are cross-sectional we are unable to determine causal relationships of the observed associations between level of FHS enrollment and USC. Nevertheless the observed associations were consistent and in the same positive direction as in other regions of the country.

## Conclusions

The results of this study have important implications for the health care model in Brazil and in other countries, especially those seeking to base their national health systems more strongly on primary health care. The study suggests that expanding and consolidating primary health care can increase access to a USC with PHC providers, as emphasized by Starfield [6] and others, assuring patients better follow-up, monitoring of chronic conditions, and attending to patient overall health needs.

## Abbreviations

CHW: community health workers
CONEP: National Commission of Ethics in Research
FHP: Family Health Program
IBGE: The Brazilian Institute of Geography and Statistics
PHC: Primary health care
PNS: Pesquisa Nacional de Saúde
SUS: Sistema Único de Saúde
USC: Usual Source of Care
